# Oxidized Low-Density Lipoprotein Serum Concentrations and Cardiovascular Morbidity in End Stage of Renal Disease †

**DOI:** 10.3390/jcdd5030035

**Published:** 2018-06-21

**Authors:** Vaia Raikou, Vasilios Kardalinos, Despina Kyriaki

**Affiliations:** 1 Department of Nephrology, DOCTORS’ Hospital, 26 Kefallinias, Athens 11257, Greece; 2 Department of Cardiology, DOCTORS’ Hospital, Athens 11257, Greece; Wolfbad@otenet.gr; 3 Department of Nuclear Medicine, General Hospital “LAÏKO”, Athens 11527, Greece; dkyriaki@gmail.com

**Keywords:** lipoprotein oxidation, oxidative stress, hemodiafiltration, cardiovascular disease, malnutrition, vitamin D

## Abstract

Introduction: Oxidized low-density lipoprotein (ox-LDL) is considered a main biomarker of oxidative stress, a common characteristic in end stage renal disease. We examined the relationship between ox-LDL serum concentrations and cardiovascular disease in permanent hemodiafiltration therapy patients. Methods: Ox-LDL values were measured by ELISA and were corrected for LDL-cholesterol (LDL-C) in 96 participants and in 45 healthy control subjects. We performed chi-square tests and adjusted models for the role of ox-LDL on cardiovascular morbidity including coronary artery disease, left ventricular hypertrophy, systolic, diastolic dysfunction and peripheral arterial disease. Results: ox-LDL/LDL-C values were significantly higher in patients than in control group (*p* = 0.02), due to increased ox-LDL serum levels rather than to low LDL-C. The unadjusted relationship between high ox-LDL/LDL-C and low ejection fraction was found significant (x^2^ = 9.04, *p* = 0.003), although the association with the other cardiovascular manifestations was found non-significant. In the adjusted model for the prediction of systolic cardiac dysfunction, high ox-LDL/LDL-C, old age and non-administration of vitamin D supplementation during dialysis session were found to be significant predictors after adjustment to the confounder. Moreover, the association between systolic cardiac dysfunction and non-administration of vitamin D derivatives during dialysis sessions was found significant (x^2^ = 6.9, *p* = 0.008). Conclusions: This study showed a significant association between high ox-LDL and systolic cardiac dysfunction in permanent hemodiafiltration therapy patients. This relationship seems to be influenced by aging and pharmaceutical therapy during dialysis sessions, including vitamin D derivatives.

## 1. Introduction

Renal disease results in accelerated atherosclerosis and increased morbidity and mortality, mainly for cardiovascular causes due either to uremia-related risk factors, such as anemia, hyperparathyroidism, inflammation, oxidative stress and malnutrition [1], or to traditional ones, such as age, male gender, diabetes, obesity, hypertension, smoking, and dyslipidemia [2]. In spite of the initiation of renal replacement therapy, the mortality remains high, above 20% per year, with more than one half of the deaths being related to cardiovascular disease [3].

Cardiovascular disease is mainly characterized by vascular occlusive disease defined by myocardial infarction and cerebrovascular strokes. The underlying disease progresses through various mechanisms, including left ventricular hypertrophy, plaque formation, arterial stiffening and endothelial dysfunction [4]. However, heart failure and sudden death are more common in these patients than atherosclerotic events.

Oxidative stress plays a significant role in the pathogenesis of cardiovascular disease, including heart failure (HF) [5]. Several plasma biochemical markers of oxidative stress were found increased in HF subjects, including oxidized low-density lipoprotein (ox-LDL) levels. Ox-LDL was suggested to play a key role in atherosclerosis development and increased plasma levels of ox-LDL were considered to be a prognostic indicator of mortality in the general population with congestive HF [6].

End-stage renal disease results in atherogenic diathesis, which is mainly associated with oxidative stress and inflammation [7]. Lipid abnormalities, including lipid oxidation, related to a dysfunctional high-density lipoprotein (HDL) may provide a role in clinical outcomes of patients in hemodialysis therapy [8].

In this study, we considered the relationship between ox-LDL serum concentrations and the development of cardiovascular disease in patients attending for permanent outpatient hemodiafiltration therapy. The preliminary hypothesis-driven for this study was based on the research regarding significant risk factors for cardiovascular morbidity and mortality in these patients.

## 2. Methods

### 2.1. Patients

This is a dual-center, retrospective and cross-sectional study of a cohort of 96 patients. The study was approved by the “Laiko, University General Hospital of Athens” and Renal Unit of “Diagnostic and Therapeutic Center of Athens Hygeia SA” Institutional Review Board. All participants provided informed oral consent prior to study enrollment.

All procedures were in accordance with the ethical standards of the institutional and/or national research committee and with the 1964 Helsinki declaration and its later amendments or comparable ethical standards.

62 males and 34 females were included in this study, on mean age 62.1 ± 14.3 years old. The median time in hemodiafiltration treatment was 5 years ± interquartile range 2.1–9.

Patients < 18 years of age at initiation of dialysis and patients who had less than 6 months of follow-up were excluded from our study. We also excluded patients with autoimmune diseases, malignancy, infections, subjects without regular vascular hemodialysis access and the patients with interdialytic weight gain of more than 5% of total body weight. Other exclusion criteria included multiple intradialytic hypotensive episodes, cerebrovascular disease, atrial fibrillation and the need to change blood pressure medications.

Participating subjects were attending for outpatient on-line-pre-dilution hemodiafiltration (on-lHDF) therapy, which was prescribed for 3-times weekly with a dialysis time of 4 h per session, a filter of 1.5 m^2^ surface area by the high-flux same material synthetic membrane, defined by an ultra-filtration coefficient >20 mL/h [9], using the same volume of replacement liquid, equal to 20 L, and a blood flow of 400 mL/min. We also used the same sodium concentration, equal to 140 mmol/L, during dialysis sessions. A bicarbonate-based ultrapure buffer dialysis solution was used with a dialysate flow rate of 500–600 mL/min, a calcium concentration of 1.50–1.75 mmol/L and low molecular weight heparin as anticoagulant therapy. The final concentration of bicarbonate in dialysate was 32 mmol/L. Dialysis sufficiency was defined by spKt/V/day (single pool, K, dialyzer clearance; t, time; V, urea distribution volume), which was calculated according to the formula of Daugirdas [10]. Patients were excluded if they had a spKt/V of less than 1.2.

The included patients were in a good status and they did not have interdialytic peripheral edema, interdialytic orthostatic hypotension or other characteristics of an inaccurate dry body weight.

The patients with pre-dialysis blood pressure ≥140/90 mmHg (*n* = 40, a prevalence of 41.7%) were considered hypertensive, and each of them received anti-hypertensive medications, including calcium channel blockers, beta-blockers or inhibitors of angiotensin II receptors. Twenty-one of the studied patients were current smokers (a prevalence of 21.9%), but none of them had alcohol intake. Thirty-five of the enrolled patients (36.5%) preserved a residual renal function (RRF) with an interdialytic urine volume of more than 100 mL.

Cardiovascular morbidity was defined as the presence of left ventricular hypertrophy (LVH, *n* = 54, 56.3%), coronary artery disease (CAD, *n* = 30, 31.3%), congestive heart failure (CHF) and peripheral arterial disease (PAD, *n* = 39, 40.6%). The coronary syndrome was documented by history of myocardial infarction, coronary artery angioplasty or bypass surgery, or clinical signs of angina pectoris. The congestive heart failure was defined by systolic dysfunction (*n* = 26, 27.1%) or by the presence of diastolic dysfunction (*n* = 67, 69.8%). The systolic function of the left ventricle (LV) was assessed by the measurement of ejection fraction (EF) and systolic dysfunction was defined as EF < 50%. The diastolic function of LV was assessed by determination of the maximum velocity of the early (E) and late (A) phase of ventricular filling and the calculation of the E/A ratio (ratio of early-to-late trans-mitral flow velocity). The diastolic dysfunction of LV was defined as E/A ≤ 1.

Peripheral arterial disease was documented by both measurement of ankle-brachial blood pressure index (ABI) and a set of clinical criteria including cool skin, reduced or lost ankle pulse on physical examination, symptomatic intermittent claudication, and by a history of past ischemic amputation or limb artery revascularization. The first and the current cardiovascular events during the study were recorded as one event for the studied cardiovascular manifestations.

Calcium-free phosphate binders were prescribed. None of the enrolled patients was receiving statin or NaHCO_3_ per os. Forty-seven of our subjects (a ratio of 49%) were being treated by intravenous administration of vitamin D supplementation by using calcitriol or paricalcitol during dialysis session, depending mainly on serum calcium and phosphate levels. None of the enrolled patients had received intravenous administration of iron during the last 2 months before the start of the study. The total of enrolled subjects was treated with erythropoietin-α or-β agent in combination to orally receiving trivalent iron (ferric hydroxide polymaltose complex).

In our data, renal failure was caused by hypertensive nephrosclerosis at a ratio of 32.3%, chronic glomerulonephritis at a ratio of 28.1%, polycystic disease at a ratio 12.5%, diabetic nephropathy at a ratio equal to 11.5% and other causes at a ratio 15.6%.

### 2.2. Blood Collection

Blood was drawn at the same time point for each subject included in the study, just before the start of the mean weekly dialysis session in a 12 h fasting state from the vascular access. The received samples were centrifuged and the fresh serum was used for the biochemical measurements of the study. Serum was also conserved and processed for various assays.

### 2.3. Laboratory Measurements

Albumin, glucose, calcium (Ca) corrected for the albumin levels, phosphate (P), cholesterol, triglycerides, low density lipoproteins (LDL) and high-density lipoproteins (HDL) were measured using spectrophotometric technique by Chemistry Analyzer (MINDRAY BS-200, Diamond Diagnostics, Holliston, MA, USA). The LDL/HDL ratio and the Ca × P products were calculated. Hematological analyzer (Sysmex, xt-4000i, Roche, Wiesbaden, Germany) was used for hemoglobin.

Oxidized LDL (ox-LDL) serum concentrations were measured using enzyme linked immunoabsorbed assay (ELISA), Immundiagnostik AG., Stubenwald-Allee (Bensheim, Germany), in accordance to manufacturer’s specification. OxLDL values were measured in the enrolled subjects and in 45 healthy subjects, who were matched for age, gender and BMI with the group of patients. The ratio of ox-LDL/LDL-C was calculated. High-sensitivity C-reactive protein (hsCRP), monocyte chemoattractant protein-1 (MCP-1) and tumor necrosis factor-a (TNF-a) serum concentrations were also measured using ELISA, Immundiagnostik AG., Bensheim, Germany, Alpco Diagnostics, Anachem, Salem, NH, USA and Ani Biotech Oy, Orgenium, Helsinki, Finland, respectively, according to the specifications of the manufacturers. The concentrations of intact-parathormone (i-PTH) and insulin were measured by radioimmunoassays (CIS bio international, Saclay, France and BioSource Europe SA, Nivelles, Belgium, respectively). Insulin resistance was calculated using the homeostasis model assessment of insulin resistance (HOMA-IR) [11].

Normalized protein catabolic rate for dry body mass (nPCR) was calculated from the urea generation rate [12]. Body mass index (BMI) was obtained from height and post-dialysis body weight.

### 2.4. Hemodynamic and Echo-Cardiographical Measurements

The mean of 12 measurements of pre-dialysis peripheral systolic and diastolic blood pressures (SBP and DBP respectively) was recorded during a treatment month using an automatic sphygmomanometer OMRON M4-I (Co Ltd. Kyoto, Japan). Mean peripheral pre-dialysis BP (MBP) was calculated as: MBP = DBP + 1/3 (SBP − DBP).

Hemodynamic and echo-cardiographical measurements were also performed before the mid-week dialysis session in combination with blood tests after resting of the patients for at least 10 min. Arterial stiffness was measured as carotid-femoral pulse wave velocity (c-f PWV) and carotid augmentation index (Aix) using the SphygmoCor system^®^ (AtCor Medical Pty. Ltd., Sydney, Australia) according to manufacturer’s specifications. In each subject, two sequences of measurements were performed, and the mean value was used for statistical analysis. Pulse pressure (PP) was derived. Ankle-brachial blood pressure index (ABI) was also calculated as the ratio of the lower values of ankle systolic pressure (pre or post-tibial artery), divided by stabilized arm systolic pressure. ABI values less than 0.9 were rated as low indicating peripheral arterial disease (PAD) and values more than 1.4 were rated as high.

Echo-cardiographical assessment was conducted by a Hewlett Packard SONOS 2500 device using a 2.25 MHz transducer (Med-Electronics, Beltsville, MD 20705, USA). All subjects were examined with the method of conventional M-mode and two-dimensional echocardiography by two cardiologists for the estimation of the hypertrophy of LV, systolic and diastolic dysfunction and of the ischemic findings according to the recommendations of the American Society of Echocardiography [4]. The left ventricular hypertrophy was defined by an inter-ventricular septum thickness more than 11.5 mm.

### 2.5. Data Analysis

Data were analyzed using SPSS 15.0 statistical package for Windows (SPSS Inc., Chicago, IL, USA) and expressed as mean ± standard deviation or as median value (±inter-quartile range) for data that showed skewed distribution. Differences between mean values were assessed by using unpaired *t*-test for two groups and data that showed skewed distributions were compared with Mann-Whitney *U*-test. Correlations between variables were defined by Spearman coefficient and the relationships between categorical variables were defined by chi-square tests. *p* values less than 0.05 were considered significant. ROC curve analysis was used for the determination of ox-LDL cut off point related to systolic cardiac dysfunction. We built models by using logistic regression analysis examining the role of ox-LDL on cardiovascular disease manifestations adjusting for traditional and specific covariates for hemodialysis patients.

## 3. Results

We corrected the measured by ELISA ox-LDL serum concentration for serum LDL-C with the calculation of the ratio ox-LDL/LDL-C.

In Table 1, the differences between patients and healthy control subjects are shown. We did note that ox-LDL/LDL-C significantly differed between patients and control group, due mainly to a significantly higher ox-LDL in combination with lower LDL-C in the group of patients in comparison to healthy control subjects.

Bivariate correlations showed that ox-LDL was significantly associated with both nPCR and serum albumin (r = 0.352, *p* = 0.001 and r = 0.204, *p* = 0.04 respectively).

The enrolled subjects were divided in two groups according to the median value of ox-LDL/LDL-C equal with 88.8 ng/mg (inter-quartile range 58.3–161.7 ng/mg).

Categorical associations showed significant relationship between high value of ox-LDL/LDL-C and systolic cardiac dysfunction (x^2^ = 7.6, *p* = 0.005), although the association with coronary artery disease, diastolic dysfunction, left ventricular hypertrophy, hypertension and peripheral vascular disease, was found non-significant.

**Figure 1:**
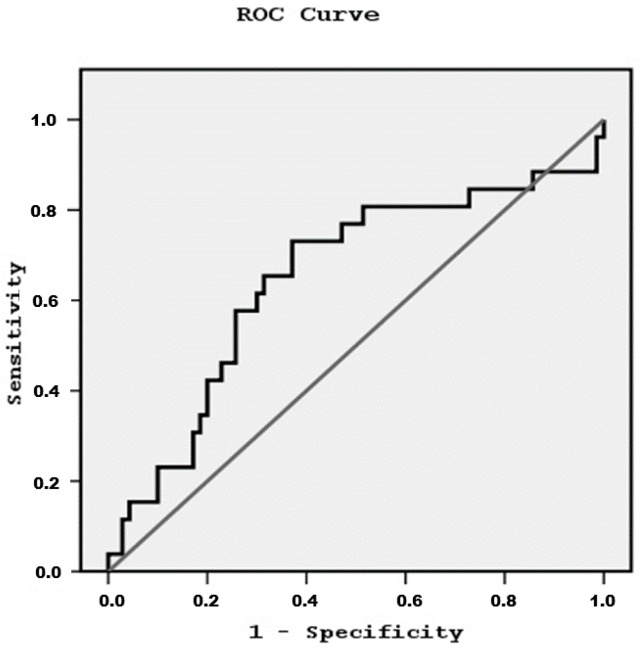
ROC curve showing the cut-off point of ox-LDL/LDL-C value related to systolic cardiac dysfunction equal to 93.07 ng/mg in enrolled subjects (*n* = 96).

**Figure 2:**
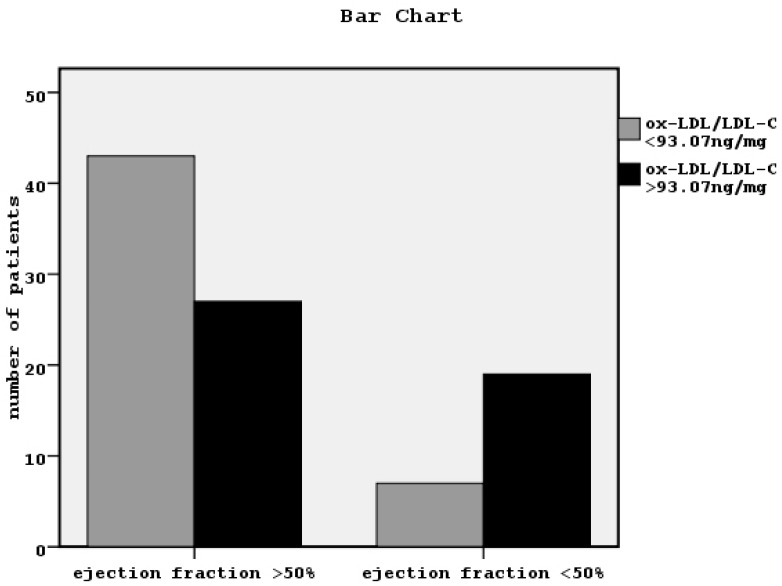
Bar Chart showing the relationship between systolic cardiac dysfunction, defined by ejection fraction less than 50% and the ratio of ox-LDL/LDL-C more or less than the cut-off point value equal to 93.07 ng/mg in our patients (*n* = 96) (x^2^ = 9.04, *p* = 0.003).

Secondarily, we divided our patients into two groups according to the cut-off point value of ox-LDL/LDL-C related to systolic cardiac dysfunction equal to 93.07 ng/mg defined by ROC curve analysis (Figure 1). x^2^ tests showed significant association between high ox-LDL/LDL-C and systolic cardiac dysfunction (x^2^ = 9.04, *p* = 0.003, Figure 2), although the association with the other cardiovascular manifestations was found non-significant.

In Table 2, the differences between the groups of patients with ejection fractions less or more than 50% are shown.

In the logistic regression analysis model built for the prediction of systolic cardiac dysfunction, high ox-LDL/LDL-C, old age and non-administration of vitamin D supplementation during dialysis sessions were found to be significant predictors after adjustment to confounders (Table 3). Moreover, the unadjusted association between systolic cardiac dysfunction and non-administration of vitamin D supplementation during dialysis sessions was found significant (x^2^ = 6.9, *p* = 0.008).

The association between non-administration of vitamin D supplementation during dialysis session and high ox-LDL/LDL-C was found non-significant. However, the patients receiving vitamin D derivatives had significantly lower ox-LDL than and similar LDL-C serum levels to the patients without vitamin D therapy (mean-Rank 41.4 vs. 55.2, *p* = 0.01 and 92.1 ± 31.2 vs. 88.9 ± 42.3, *p* = 0.7, respectively).

## 4. Discussion

In this study, we observed significant association between high ox-LDL corrected to LDL-cholesterol serum concentration and systolic cardiac dysfunction.

The patients in hemodialysis are highly subjected to increased oxidative stress and inflammation, either by their own characteristics, such as advanced age and high prevalence of diabetes mellitus, or by characteristics related to uremia and dialysis therapy, such as dialysis vintage, biocompatibility of dialysis membranes, exposure to endotoxins, parenteral administration of iron and the usage of erythropoiesis agents [13]. In this study, the participating patients were being treated using the same material dialysis membrane in every dialysis session, nobody had been treated by statin or parenteral administration of iron during the time period before study enrollment, and everybody was regularly receiving erythropoiesis agents in combination with orally receiving iron.

However, we observed elevated values of ox-LDL/LDL-C in our patients compared to healthy control subjects, who were matched for the age, gender and BMI. We also noted that the group of patients had significantly lower total cholesterol, HDL-C and albumin values as markers of nutritional status, although they had significantly higher triglycerides, hsCRP, i-PTH, insulin, insulin resistance, TNF-a, ox-LDL and mildly lower LDL-C in comparison with control group. The elevated markers of malnutrition in conjunction to inflammatory markers result in the co-occurrence of malnutrition, inflammation, and atherosclerosis (MIA syndrome) in dialysis patients, in agreement with the characteristics of our subjects, in spite of our exclusion criteria and the good status of our participants. Malnutrition has already been reported as a specific cardiovascular risk factor for this population of patients, and each of the MIA syndrome components worsens the survival of these patients [14]. Moreover, in this study we observed a significantly positive association between ox-LDL and both nPCR and serum albumin. By these findings, we could observe that the increased ox-LDL/LDL-C ratio in our patients comparative to control group was mainly based on high ox-LDL caused by oxidative state, and less on low LDL-C caused by malnutrition. We also could support that oxidative status in our data may be attributed to metabolic disorders due to uremia resulting in oxidative stress rather than to the conditions of hemodiafiltration therapy.

On the other hand, previously, it has been reported that oxidative stress is included into major determinants of metabolic disorders such as insulin resistance in hemodialysis patients, in combination with excess visceral fat and inflammation [15,16]. In this study, our patients had higher insulin and insulin resistance defined by HOMA-IR connected to higher ox-LDL than the healthy control group.

Oxidative stress can be defined as a tissue injury resulting from an imbalance between the generation of oxidizing components and insufficient production of antioxidant mechanisms [17]. Previously, it has been reported a significant correlation between oxidative stress markers, such as plasma glutathione, superoxide dismutase (SOD) and malonyldialdehyde (MDA) levels and cardiac dysfunction, including low ejection fraction in the end stage of renal disease [18]. Regarding the role of ox-LDL, it has been demonstrated that elevated levels of ox-LDL and ox-LDL autoantibodies in the plasma were associated with a lower ejection fraction in general population [19]. Moreover, a recent study showed increased ox-LDL levels in the left ventricle cavity, rather than in peripheral blood, suggesting an important contribution by localized ox-LDL formation in heart failure conditions [20].

In maintenance hemodialysis patients, previous reports showed no association between ox-LDL and major adverse cardiac events [21]. However, in chronic kidney disease patients, it has been suggested that ox-LDL may be a valuable marker of oxidative stress correlated with left ventricular hypertrophy [22]. In contrast, in this study, we did not observe such a significant association, although we noted significant association between high ox-LDL/LDL-C and low ejection fraction. These controversial findings may request bigger studies looking upon such an object.

Examining the groups of our subjects with and without low ejection fraction, we observed that those with low EF were elder and they had significantly elevated arterial stiffness markers, such as c-f PWV, Aix and PP, thickness of left ventricle wall and characteristic of diastolic dysfunction defined by low E/A ratio in combination to higher ox-LDL, ox-LDL/LDL-C ratio and hsCRP than the patients with EF more than 50%.

In combination with the important role of MIA syndrome, it has been established that arterial stiffness and vascular calcification are non-traditional cardiovascular risk factors in chronic kidney disease resulting in coronary arterial disease and heart failure [23]. Vascular calcification requires differentiation of vascular smooth muscle cells into osteoblast-like cells. This phenomenon can be activated by inflammatory cytokines and production of reactive oxygen species (ROS) [24]. The increased ROS generation during oxidative stress leads to more inflammation, since ROS are released from monocytes and polymorphonuclear cells with pro-inflammatory cytokines, which, in turn, amplify the generation of oxidants [25].

The presence of MIA syndrome related to arterial stiffness and vascular calcification may be included in pathophysiological mechanisms which outline the relationship between high ox-LDL and systolic cardiac dysfunction in this study. The built adjusted model also supported the importance of elevated ox-LDL in conjunction to aging and non-administration of vitamin D during dialysis session for the prediction of systolic dysfunction. Furthermore, we observed a significant unadjusted association between low ejection fraction and non-administration of vitamin D supplementation during dialysis sessions, and the subjects who were regularly receiving vitamin D derivatives had significantly lower ox-LDL than the patients who were not receiving vitamin D supplementation, although they had similar LDL-C.

In agreement, recently it has been reported that the usage of vitamin D derivatives in chronic kidney disease patients may prevent the elevated ROS production and the cytokine up-regulation by serum phosphate and may decrease the tissue factor expression in smooth muscle cells, resulting in beneficial cardiovascular effects [26,27].

## 5. Conclusions

This study showed a significant association between high ox-LDL and systolic cardiac dysfunction in permanent hemodiafiltration therapy patients. This relationship seems to be influenced by the aging and the pharmaceutical therapy during dialysis session including vitamin D derivatives.

### Limitations

The important limitation of the study is its cross-sectional nature, which prevents the definition of the relationship between cause and effect in combination with the small number of included subjects. Also, the measurement of other markers of oxidative stress was unavailable.

## Figures and Tables

**Table 1:** Differences between hemodiafiltration patients (*n* = 96) and healthy control subjects (*n* = 45) (* *p* < 0.05).

Characteristic	Hemodiafiltration Patients (*n* = 96) Mean ± SD or/Mean Rank	Healthy Control Subjects (*n* = 45) Mean ± SD or/Mean Rank	*p* Value
Gender (males %/females %)	62(64.6%)/34(35.4%)	24 (53.3%)/21 (46.7%)	0.1
Age (years)	62.1 ± 14.2	59.6 ± 11.9	0.6
BMI (Kg/m^2^)	25.08 ± 3.9	25.9 ± 3.6	0.2
Cholesterol (mg/dL)	163.4 ± 45.3 *	188.7 ± 27.3	0.001
LDL (mg/dL)	90.5 ± 37.2	99.08 ± 29.6	0.8
HDL (mg/dL)	38.9 ± 9.7 *	53.9 ± 8.9	0.001
Triglycerides (mg/dL)	172.1 ± 87.1 *	86.8 ± 35.6	0.001
oxLDL (ng/mL)	69.4 *	54.4	0.03
OxLDL/LDL (ng/mg)	76.2 *	59.8	0.02
Glucose (mg/dL)	96.2 ± 22.2 *	89.06 ± 9.6	0.009
Insulin (μU/mL)	78.6 *	50.8	0.001
HOMA-IR (mmol/L)	79.9 *	52.02	0.001
hsCRP (mg/L)	8.6 ± 5.9 *	2.8 ± 2.3	0.001
MCP-1 (pg/mL)	69.8	68.6	0.9
TNF-α (pg/mL)	83.6 *	65.08	0.01
i-PTH (pg/mL)	86.08 *	34.09	0.001

**Table 2:** Differences between groups of patients with and without systolic dysfunction, defined by ejection fraction less or more than 50% in a total of 96 hemodiafiltration patients (* *p* < 0.05).

Characteristic	Patients with Ejection Fraction Less Than 50% (*n* = 26) Mean ± SD/Mean Rank	Patients with Ejection Fraction More Than 50% (*n* = 70) Mean ± SD/Mean Rank	*p* Value
Age (years)	68.8 ± 12.6 *	59.6 ± 14.1	0.005
Dialysis vintage (years)	52.8	46.9	0.4
spKt/V for urea	46.1	49.4	0.6
nPCR (g/Kg/day)	2.2 ± 0.6	2.2 ± 0.5	0.9
Interdialytic urine volume (mL)	225 ± 154.1	361.4 ± 326.4	0.3
Interdialytic weight gain (L)	2.2 ± 0.7	2.3 ± 1.1	0.4
BMI (Kg/m^2^)	24.8 ± 3.3	25.2 ± 4.04	0.7
Hb (gr/dL)	11.6 ± 1.3	11.9 ± 1.4	0.3
Cholesterol (mg/dL)	158.5 ± 47.3	165.2 ± 44.8	0.5
LDL (mg/dL)	85.2 ± 35.1	92.5 ± 37.8	0.4
HDL (mg/dL)	38.5 ± 10.6	39.02 ± 9.4	0.8
Triglycerides (mg/dL)	181.7 ± 111.6	168.6 ± 76.7	0.6
oxLDL (ng/mL)	55.9	45.7	0.1
OxLDL/LDL (ng/mg)	58.9 *	44.6	0.02
Albumin (gr/dL)	43.8	50.2	0.3
Glucose (mg/dL)	95.03 ± 20.9	96.6 ± 22.9	0.7
Insulin (μU/mL)	44.7	49.9	0.4
HOMA-IR (mmol/L)	44.9	49.8	0.5
hsCRP (mg/L)	12.06 ± 5.4*	7.4 ± 5.7	0.001
MCP-1 (pg/mL)	51.3	41.04	0.1
TNF-α (pg/mL)	50.06	44.3	0.3
i-PTH (pg/mL)	47.3	48.9	0.8
SBP (mmHg)	135.1 ± 24.6	130.4 ± 23.5	0.4
MBP (mmHg)	99.4 ± 14.8	97.2 ± 13.6	0.5
c-fPWV (m/s)	12.04 ± 1.9 *	11.02 ± 1.7	0.01
Augmentation index (Aix, %)	25.04 ± 2.2	23.8 ± 2.3	0.03
Pulse pressure (PP, mmHg)	64.5 ± 22.7 *	54.4 ± 19.1	0.03
ABI	±0.5	±0.4	0.4
EF (%)	25.8 ± 2.7 *	58.4 ± 3.07	0.001
E/A ratio	31.5 *	54.8	0.001
Thickness of interventricular septum (mm)	13.7 ± 2.0 *	12.1 ± 2.0	0.001

**Table 3:** Logistic regression analysis for the prediction of systolic cardiac dysfunction using traditional and specific risk factors for hemodiafiltration patients.

Characteristic	*p*-Value	Odds Ratio	Confidence Interval
age	0.01	1.06	1.01–1.11
gender	0.8	1.1	0.3–3.8
Diabetes mellitus	0.6	1.7	0.2–11.3
hypertension	0.1	0.4	0.1–1.4
smoking	0.4	1.9	0.4–8.6
spKt/V for urea	0.9	0.9	0.2–4.5
nPCR	0.2	0.5	0.2–1.5
Vitamin D therapy	0.02	0.2	0.06–0.8
Ox-LDL/LDL-C	0.004	6.2	1.8–21.2
